# Cohort Profile: The Kilifi Vaccine Monitoring Study

**DOI:** 10.1093/ije/dyw202

**Published:** 2016-10-26

**Authors:** Ifedayo MO Adetifa, Tahreni Bwanaali, Jackline Wafula, Alex Mutuku, Boniface Karia, Anne Makumi, Pauline Mwatsuma, Evasius Bauni, Laura L Hammitt, D James Nokes, Ephantus Maree, Collins Tabu, Tatu Kamau, Christine Mataza, Thomas N Williams, J Anthony G Scott

**Affiliations:** 1 Epidemiology and Demography Department, KEMRI-Wellcome Trust Research Programme, Kilifi, Kenya; 2 Department of Infectious Diseases Epidemiology, London School of Hygiene and Tropical Medicine, London, UK; 3 Department of International Health, Johns Hopkins Bloomberg School of Public Health, Baltimore, MD, USA; 4 School of Life Sciences and WIDER, University of Warwick, Coventry, UK; 5 Unit of Vaccines and Immunisation Services; 6 Vector Borne Diseases Control Unit, Ministry of Health, Nairobi, Kenya; 7 County Department of Health, Kilifi, Kenya; 8 Department of Medicine, Imperial College, St Mary's Hospital, London, UK; 9 INDEPTH Network, Accra, Ghana

## Why was the cohort set up?

Childhood vaccination programmes have significantly reduced childhood morbidity and mortality.^1^ Since 2000, there has been an unprecedented expansion of routine childhood vaccination and increased access to new vaccines in developing countries.^2^^,^^3^ Vaccines protect the individual recipient (direct protection) but they may also protect the whole population (indirect protection) if they interrupt the chain of transmission of the target disease.^4^^,^^5^ Good quality population- and-individual level epidemiological data are needed to estimate direct and indirect effects and inform vaccination policy at the national level. To assure society that a vaccine programme is safe, it is also necessary to monitor for adverse events following immunization (AEFI).

During the introduction and expansion of access to new vaccines in low- and middle-income countries (LMICs), relatively few investments are allocated to evaluation of the impact and cost effectiveness of vaccination programmes, which is required to achieve long-term sustainability of new vaccine programmes in LMICs. The capacity for these kinds of impact assessments has lagged significantly behind the introduction of new vaccines. As a result, only a very small number of low-income countries have the platforms required to assess vaccine impact, effectiveness and safety. Some countries have national or subnational platforms for monitoring vaccine coverage, e.g. in Health and Demographic Surveillance Systems, periodic multi-indicator cluster surveys and Demographic and Health Surveys (DHS). Although these can be linked to mortality surveillance in HDSS sites to determine the population effects of vaccines, data quality and interpretation are limited.

The Kenya Medical Research Institute-Wellcome Trust Research Programme (KWTRP) in Kilifi set up the *Haemophilus influenzae type b* (Hib) conjugate vaccine effectiveness study in 2000. It was further expanded in 2008 with addition of the real-time vaccine monitoring component, in anticipation of the introduction of pneumococcal conjugate vaccine (PCV) in Kenya. The objective of the Kilifi Vaccine Monitoring Study (KiVMS), a long-term continuous cohort study, is to investigate effectiveness, impact, coverage, safety and indirect vaccine effects by recruiting birth cohorts as well as cohorts of older children and adults where applicable, within a well-characterized population and area. In addition, KiVMS is used to explore the determinants of vaccine coverage and acceptability in the population. Built on the platform of a Health and Demographic Surveillance System (HDSS), KiVMS integrates morbidity surveillance systems at the County Department of Health (CDOH), Kilifi, and a population-based, computerized information system for collecting vaccination data. Therefore it has the following essential attributes: continuously updated demographic data from the population of interest (e.g. births, deaths and migration); and complete and accurate vaccination records for the catchment population. Vaccine information systems are rare in tropical Africa.

Here we describe the study population and provide an overview of the data sources and data management processes.

## Who is in the cohort?

### Setting

**Figure 1:**
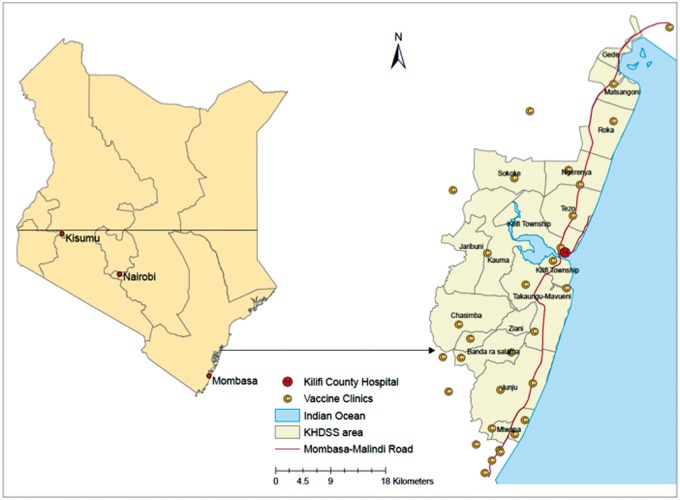
Areas and facilities covered by the Kilifi Vaccine Monitoring Study.

Kenya is divided administratively into 47 counties.^6^^,^^7^ Kilifi County, on the Indian Ocean Coast, is one of the poorest^6^ and is typical of a rural equatorial Africa setting. KiVMS is based in Kilifi, with the area covered by the Kilifi HDSS (KHDSS) as shown in Figure 1. The KHDSS has a population of 280 000 covering an area of 891 km^2^.^8^

### Inclusion

The primary target of this study is the population of children aged < 5 years, resident in the study area. The KHDSS has a birth cohort of ≈ 8000 per annum. In addition, all childhood immigrants are recruited, along with their families, into the KiVMS during re-enumeration rounds. From January 2011 to 31 December 2014, there were 33 962 children in the birth cohort database.

### Community engagement and governance

KiVMS was conceived at the outset as collaboration between the Ministry of Health and the KWTRP. A Memorandum of Understanding between both parties guides this collaboration. Its purpose is to support national and regional policy making by providing informative local data. In addition, this resource provides evidence to support the functions of the newly established Kenya National Immunisation Technical Advisory Group (KENITAG).

### Ethical approval

The KEMRI Scientific and Ethics Review Unit approved this study and the activities carried out on the KHDSS platform.

## What has been measured?

### Basic demographic data

Basic demographic data are obtained from the KHDSS platform. In brief, these include global information system (GIS) mapping of homestead location, household name and head, individuals, residency status, births, deaths and migration. The KHDSS is a longitudinal surveillance of the population living in a well-defined geographical area around Kilifi County Hospital (KCH), which has been updated through household visits, monitoring vital events and migration, since the year 2000.^8^

### Ascertainment of vaccination

**Figure 2:**
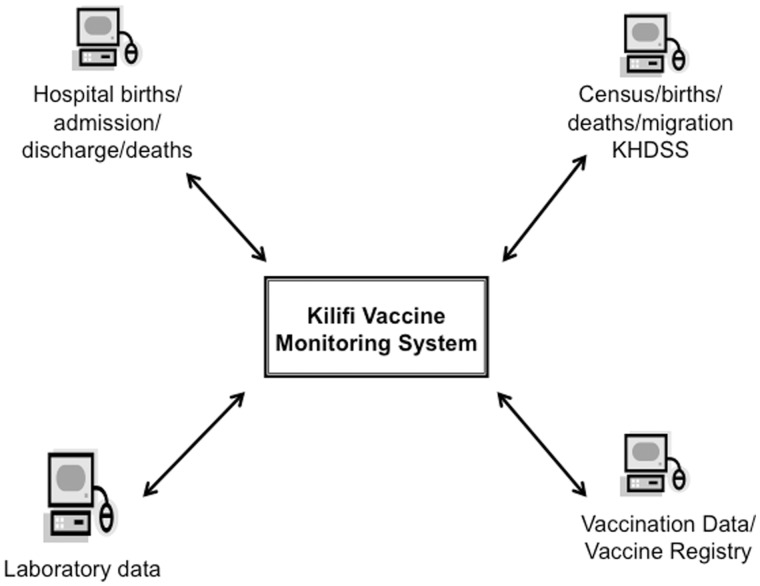
Illustration of the linkages between the constituent parts of the Kilifi Vaccine Monitoring System (KiVMS).

Using an electronic vaccine monitoring system established at all 34 health facilities delivering vaccines and 53 affiliated outreach sites in the KHDSS (Figure 1), data clerks record vaccine data (Table 1). Vaccine clinics are either government^26^ or privately^8^ owned and located within or just outside the KHDSS boundaries. Children presenting to these are matched to their unique personal record in the population register. If their details do not exist in the KHDSS database, they are registered as new once matched to the mother’s homestead and details. If they are not matched to a household, they are registered with a temporary identification pending resolution of the associated data query. Manual registers provide a source of back-up data for verification like the vaccine cards retained by mothers/caregivers, which are labelled with a unique identity number. Linkage of clinic and central server data is achieved weekly; data captured at the clinics during daily operations are uploaded to laptops brought on site by data supervisors, and the latest version of the population register is downloaded to data clerks’ laptops. The population register is also updated with data of children newly registered at the vaccine clinics and previously unknown to the KHDSS. All of the data are delivered to the central data server at the KWTRP. The synchronisation lag time is usually 1 week. The linkages between the constituent parts of the KiVMS are outlined in Figure 2.

**Table 1.** Data recorded in the Kilifi Vaccine Monitoring System

Characteristics	KiVMS database
**Attributes of the child**	Child’s name Date of birth Unique personal identifier for child’s vaccine card Unique personal identifier for child’s mother Mother and homestead data Residence, and demographic details if individual is not in the database
**Attributes of vaccine clinic visit**	Vaccine clinic visited Type of visit-for vaccination or anthropometry Data entry clerk identifier Update of all vaccines recorded in vaccine card but not seen in registry
**Attributes of vaccine**	Date of vaccination Vaccine delivered/stock out Refusals Type of vaccine and dose given Vaccine aliquot given (for multi-dose vials)
**Attributes of hospital visit**	Date of admission Duration of admission Admission and discharge diagnosis KHDSS residence status

### Morbidity surveillance

All paediatric and adult admissions undergo detailed clinical and laboratory evaluation(s) for vaccine-preventable disease surveillance at the KCH, a 172-bed (and 20-cot) facility at the centre of the KHDSS area that provides primary care and serves as a first-level referral hospital.^8^ It is equipped for basic haematological and biochemical tests and advanced microbiological culture. It also offers basic radio-diagnostic support.^12^ Records of births and maternal deaths are also recorded from the maternity section in real time. KiVMS is supported by a bespoke database and platform that integrates electronic health records at KCH with vaccination records and the KHDSS population register. Individuals at admission or delivery are matched with the population register, creating a permanent link between the patient’s residence record and the hospital event. Individuals are matched on five criteria: name, sex, date of birth, residence and homestead characteristics.

### Cross-sectional surveys

Surveys of intermediary markers of vaccine impact, such as nasopharyngeal carriage of pneumococci or serological responses to vaccine-preventable diseases, are assessed through recurrent standardized surveys by age-stratified random sampling of the entire population. These have been used to determine the interruption of transmission of pneumococci^9^ and the population immunity to Hib vaccine.^10^ In addition, we propose to validate epidemiological measures of vaccine coverage using these samples.

## How often have they been followed up?

Vaccination data are recorded at every vaccine clinic visit. Re-enumeration of births, deaths and migration events in the KHDSS occurs three times a year.^8^ Nasopharyngeal carriage studies are carried out annually and the serological surveys biannually.^9^ In addition, births are recorded continuously as they occur or at first contact in the community during re-enumeration or at clinics during vaccination visits. Morbidity surveillance at the KCH is continuous.

## What has been found? Key findings and publications

### Vaccine impact using before-after studies

The introduction, in 2001, of Hib conjugate vaccine (as pentavalent vaccine with diphtheria, tetanus, whole-cell pertussis and hepatitis B antigens) was the precipitant for the development of the KiVMS. Using population-linked morbidity surveillance, we showed an 88% effectiveness of the vaccination programme against invasive Hib disease incidence among children aged less than 5 years, within 3 years of introducing the vaccine.^11^ Fifteen years on, and without a booster dose, vaccine effectiveness is 93% and sero-surveys confirm enduring population immunity.^10^

KiVMS currently supports the Pneumococcal Conjugate Vaccine Impact Study (PCVIS), a before-after study of the impact of the 10-valent pneumococcal conjugate vaccine (PCV-10) introduced in January 2011. Linkages between the vaccine registry and morbidity surveillance databases permit an individual-based cohort analysis of the entire population by connecting rates of invasive pneumococcal disease (IPD) to vaccine status. Dividing the numbers of IPD cases by the person-years of observation in different exposure strata (unvaccinated, partially and fully vaccinated) provides estimates of the total and indirect effects of PCV-10. The impact on the incidence of clinical and radiologically confirmed pneumonia and invasive pneumococcal disease will be reported in 2016.

KiVMS was recently adapted to estimate the impact of the newly introduced rotavirus vaccine. Between 2002 and 2004, incidence of hospitalizations with Group A rotavirus gastroenteritis was 1431 [95% confidence intervals (CI])1275-1600] per 10,000 person years of observation (pyo) in infants.^12^ Ongoing surveillance shows these rates declined appreciably over time before vaccine introduction in July 2014. It is important to adjust for secular trends like these in assessments of vaccine impact, especially if this change is thought to be due to changes in associated risk factors. Rotavirus vaccination impact data will be available in 2017.

### Epidemiological studies of transmission and sero-prevalence

Following the introduction of PCV-10 with a catch-up campaign in all children aged < 5 years in the KHDSS, annual studies of nasopharyngeal carriage demonstrated a reduction of 64% (95% CI 49-74%) in the prevalence of vaccine serotype pneumococci among children aged < 5 years. There was also a 66% (95% CI 38-82%) reduction in carriage prevalence among unvaccinated older children and adults, illustrating a profound and rapid indirect protection and predicting a decline in IPD across the whole population.^9^

### Assessments of vaccine coverage, timeliness and equity

**Figure 3:**
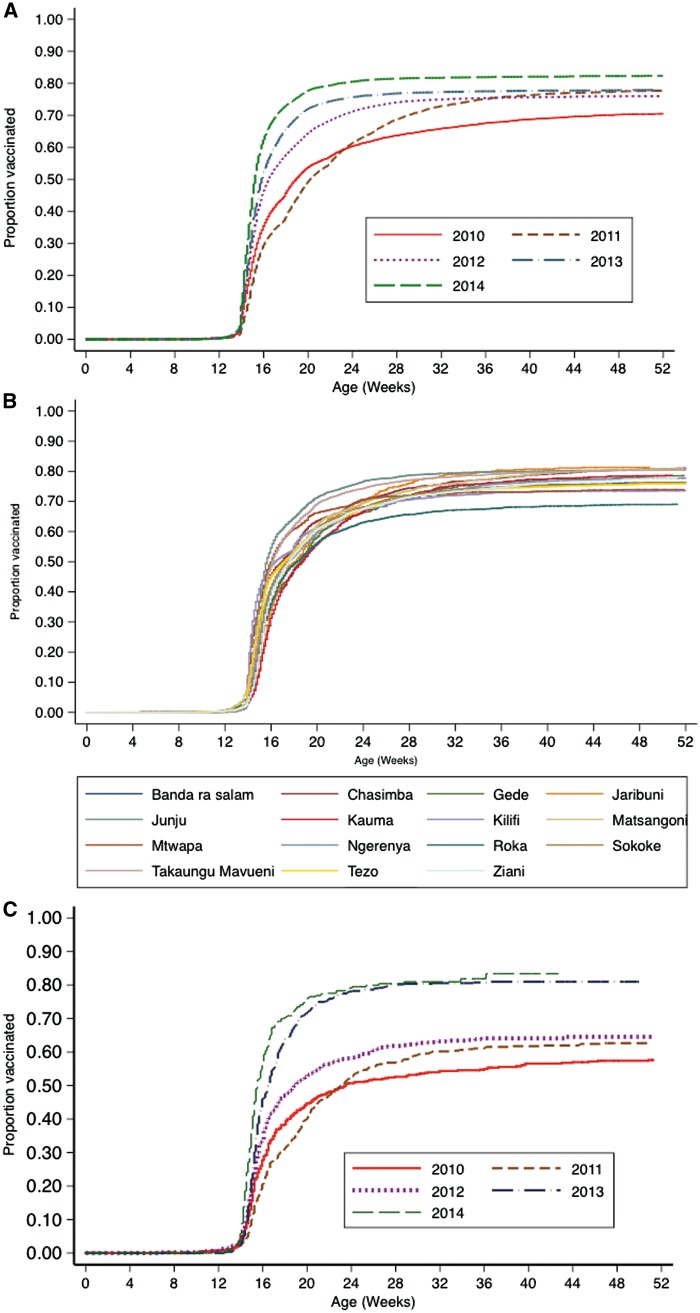
Five-year pattern of coverage and time-to-vaccination for the third dose of the DPT (pentavalent) vaccine. (A) By birth cohort in the KiVMS overall. (B) By birth cohort in all KiVMS study locations. (C) By birth cohort in one location, Roka, within the KiVMS study area.

KiVMS provides a platform to validate administrative and survey-based methods for assessing vaccine coverage. Similarly to others,^13^ we found that compared with survey data, administrative estimates exaggerate vaccine coverage.^14^ Within KHDSS, we have observed that seasonality and family size are strong factors that determine coverage.^14^^,^^15^ KiVMS allows for review of patterns of coverage over time to monitor programme performance by birth cohort and locations (Figure 3A, B); it gives insights into equity of access by its sensitivity for identification of sub-populations with low vaccination coverage (Figure 3C) and can also be used to investigate vaccine failures and target interventions. Predictors of vaccine inequity and hesitancy in at-risk groups such as recent migrants and young mothers, and in geographical pockets of poor coverage, can also be investigated.

### Complex before and after studies

**Figure 4:**
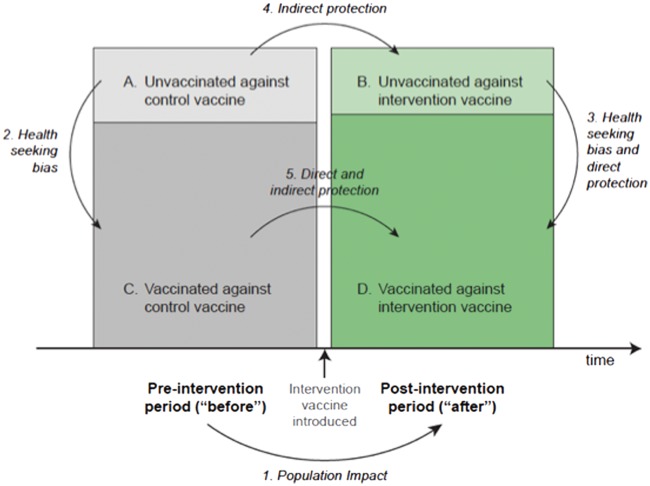
Comparisons required for estimating overall vaccine effectiveness, and direct and indirect vaccine effects.

Before-after studies and case-control studies are susceptible to similar biases. In routine practice, the population of children who are not immunized may differ from the majority with respect to background incidence or the extent to which their disease outcome can be fully ascertained. An accurate estimate of effectiveness for individual protection (direct effect) can only be obtained by adjusting for confounding by ‘healthy’ vaccinees.^16^ It is important to identify these ascertainment biases and to control for them to the extent possible, for example by estimating the protection from disease by receipt of an unrelated vaccine. The schematic shown in Figure 4 highlights the various cohort and incidence rate comparisons required to estimate the overall vaccine impact as well as the direct and indirect protection by a vaccine.

A further complexity arises from secular changes in disease risk factors. As vaccine ‘exposure’ is always associated with time in a before-after study, any risk factor that also varies with time may be a confounder. In tropical Africa, the risk factors for vaccine-preventable diseases are complex and may include malaria, malnutrition and HIV infection. Analyses of incidence ratios attributable to vaccination in an interrupted time series analyses, for example,^17^ can adjust for secular trends in major confounders but only if these data are available. By virtue of its setting within a community and hospital-based research station of over 25 years’ duration, the KiVMS has access to data on many of these variables.^18^

### Vaccine safety monitoring

KiVMS follows a relatively small annual birth cohort compared with the national immunization programme, but it has the capacity to accurately define temporal associations between recent vaccination and deaths or serious life-threatening events presenting to hospital. When the World Health Organization (WHO) considered the introduction of PCV10 as a two-dose vial without a preservative, they were concerned about the theoretical risk of bacterial contamination of an opened vial leading to AEFI after the second dose in the vial. We studied the problem for the first 2 years of introduction. The absence of any measurable safety signal in vaccination site abscesses, sepsis or death after immunization helped in the approval of PCV10 introduction into other countries using the two-dose vial.^19^

Because the mortality burden attributable to many vaccine-preventable diseases is high in sub-Saharan Africa (sSA), the issue of vaccine safety has not been the primary focus of society. However, experience from developed countries suggests that vaccines may be valued less highly once the target disease has been brought under control, and assurances of safety are essential for the sustainability of the programme.

In 2014, Kenya scaled up its maternal tetanus vaccination programme because earlier efforts and success had brought the country within range of the global maternal and neonatal tetanus elimination threshold, i.e. incidence < than 1 case per 1000 live births.^20^ Unfortunately, a group of religious leaders accused the government of planning to sterilize women by giving beta-human chorionic gonadotropin (HCG)-containing tetanus vaccines, and campaigned against this initiative.^21^ To support their position, they argued that the expanded programme was not justified because there were no more cases of neonatal tetanus in the country. However, data from Kilifi clearly showed the impact of the immunization programme and the need to build on the progress achieved already.^22^

## What are the main strengths and weaknesses?

The evaluation of population impact and safety in the diverse epidemiological settings where vaccines are introduced receives less attention compared with phase III trials to demonstrate individual vaccine efficacy. Although KiVMS has evolved to meet a specific need in Kenya, its principal strength is its unique integration of a vaccine registry and a morbidity surveillance system on top of the largest HDSS in Africa. As a cohort study and integrated surveillance platform, it facilitates population-level vaccine impact assessments. The benefits of such a set-up have recently been recognized by the INDEPTH network in its recently proposed model: the Comprehensive Health and Epidemiological Surveillance System (CHESS).^23^ It is a very efficient study template for gathering data on vaccine effectiveness and safety, which can be copied or deployed across heterogeneous locations in the developing world. It has provided evidence of direct and indirect vaccine effectiveness^9^^,^^11^ and vaccine safety,^19^ provided insights into vaccination coverage^14^^,^^15^ and facilitated cost-effectiveness analyses using models for pneumococcal,^24^ rotavirus^25^ and Hib vaccines,^26^ and thus directly influenced national and regional policy.

Vaccine monitoring is conducted in clinics entirely by CDOH staff. The KWTRP provides the design, training and data collation, cleaning and analysis. This integration with the health ministry personnel is another strength of KIVMS that has been shaped significantly by more than a decade of collaboration. This has proved essential both for the smooth running of the programme and for the effective use of results.

The KEMRI Scientific and Ethics Review Unit (SERU) approved the creation of KiVMS as part of the KWTRP. Importantly, all community-based research at the KWTRP are part of an integrated system of community engagement using a wide range of channels including community representative groups and open public meetings to ensure that the research conducted under KiVMS is locally relevant.

As expected of a resource-poor setting, there are challenges of logistics and infrastructure. The limited coverage and instability of power supplies, along with inadequate roads and mobile phone networks, present tremendous challenges. As the project did not have capacity for electronic data capture during outreach services (where healthcare workers travel intermittently to numerous alternative delivery points, e.g. schools), back-up paper systems were deployed. Supplementary immunization activities (e.g. for measles and polio) are also conducted in KHDSS communities from time to time. However, the present infrastructure of KiVMS only allows for the recording of routinely delivered vaccinations.

In KiVMS, it is critical to identify individuals accurately from the population register and link them to events such as vaccination or hospital admission. Identification is generally easier at vaccine clinics than hospitals because mothers and data clerks know the local area in detail and geographical residence is a key identity criterion. However, vaccine clinics are very busy environments and personal identity (ID) matching is still slow and occasionally inaccurate. An incident record is opened when an ID mismatch occurs, which is resolved by data supervisors and managers of the vaccine registry and KHDSS at the KWTRP. Fingerprinting technology solutions were considered but would not work for our primary target population–young infants–as their fingerprint patterns are not reliably distinguished at this age.

Although KHDSS detects in- and out-migrations in its study area, the data capture in local clinics cannot record vaccinations received by migrants if they had received all of their vaccines before moving into the area and do not visit the vaccine clinics nor experience hospitalization at KCH. In addition, migration itself may be a risk factor for poor uptake.^27^^,^^28^ Consequently, data for migrants are less complete and there is a risk of misclassification. To capture these data as far as it is practically possible, we instituted vaccine-card surveillance for KHDSS immigrants aged < 5 years, which is effectively a small population sample, during re-enumeration rounds. This will improve completeness of data for this small but often at-risk group. In Table 2, we show the merits of an electronic vaccine registry compared with use of HDSS enumeration rounds for routine collection of all vaccine data.

**Table 2.** Advantages and disadvantages of the use of a clinic-based electronic vaccine monitoring system compared with vaccine card verification during enumerations rounds in a health and demographic surveillance system

Vaccine Registry	Vaccine card survey at enumeration rounds
1. Allows for real-time monitoring vaccine coverage data	1. It cannot provide real-time vaccine coverage data
2. Facilitates rapid intervention/reaction to improve coverage and/or correct immediate problems	2. Produces data too late for directing interventions for problem solving or to improve coverage
3. More difficult to initiate but relatively easy to maintain	3. Convenient and relatively easier to set up
4. Not dependent on good record keeping and entries at vaccine clinic, but electronic platform helps improve record keeping	4. Dependent on good record keeping and entries at vaccine clinics
5. Response rate is not dependent on card retention, data are obtained at real time in vaccine clinics	5. Response rate is dependent on card retention in population
6. Risk of missing data in migrants, especially older children	6. May miss migrants but more likely to reach them with repeated cycles of data collection
7. Facilitates linkages across all vaccine clinics and electronic health records at referral hospital and for catchment population	7. Not possible to link to morbidity and other registries in real time
8. Provides more opportunities for updating vaccine records especially when linked with hospital and other records	8. Typically stand-alone and does not provide other opportunities for updating individual vaccine records
9. Less risk of non-response error and missing data	9. Increased risk of non-response error
10. Can be used or linked to other modules for increasing vaccine coverage, e.g. reminders/recall	10. Cannot be linked or extended to serve other purposes such as reminders/recall for vaccination
11. Has utility for tracking, for example bar-coded vaccine vials, and for vaccine-associated adverse events surveillance for assurance of vaccine safety	11. Contributes very little to surveillance of vaccine-associated adverse events
12. Not dependent on presence of primary caregiver	12. Dependent on presence of card holder/primary caregiver who is often not available
13. Requires more investment since it is population wide	13. Relatively cheaper when limited to a sample of the target population, like migrants in this case

KHDSS, the largest surveillance of its kind in tropical Africa, is suitable for the study of vaccine impacts against common diseases (e.g. invasive Hib and pneumococcal disease) but cannot provide the richness of detail, e.g. strain-specific or age-specific vaccine efficacy afforded by national surveillance systems. This limitation is most apparent in the study of vaccine safety, as the levels of severe AEFI for licensed vaccines are infrequent in epidemiological terms and cannot easily be associated with vaccine in a population of this size. One solution to this is to link several HDSS platforms together, within country as we have done in Kenya, to examine PCV10 safety.^19^

## Can I get hold of the data? Where can I find out more?

Investigators with interest in datasets or collaborations can contact Millicent Odhiambo [modhiambo@kemri-wellcome.org] and the KWTRP data governance committee [dgc@kemri-wellcome.org] with a statement of request and formal application for data transfer. In addition, they can contact the principal investigator, Professor Anthony Scott [ascott@kemri-wellcome.org] and/or co-investigator, Dr Ifedayo Adetifa [IAdetifa@kemri-wellcome.org]. There is more information on the KWTRP website [www.kemri-wellcome.org].

Profile in a nutshell

- The Kilifi Vaccine Monitoring Study (KiVMS) is a long-term continuous cohort study set up to investigate effectiveness, impact, coverage, safety and indirect vaccine effects by recruiting birth cohorts and, where applicable, cohorts of older and adults.
- It is based in the area covered by the Kilifi Health and Demographic Surveillance System, Kilifi, Kenya, and currently has records of 33 962 children in the birth cohort database.
- A major strength of KiVMS is its unique integration of a vaccine registry, a morbidity surveillance system and the largest health and demographic surveillance system (HDSS) in Africa.
- Requests for data and/or collaboration should be sent to [dgc@kemri-wellcome.org and MOdhiambo@kemri-wellcome.org]

## Funding

KiVMS is funded by a number of sources, notably Gavi, the Vaccine Alliance and the Wellcome Trust. J. Anthony G. Scott, Thomas N. Williams. and D. James Nokes are funded through fellowships from the Wellcome Trust (098532, 091758 and 090853, respectively)
